# Draft Genome Sequence of *Escherichia coli* E1728 Isolated from Marine Sediment in Hong Kong

**DOI:** 10.1128/genomeA.00430-14

**Published:** 2014-05-08

**Authors:** Jennifer Y. H. Lai, Hao Zhang, Miranda H. Y. Chiang, Min Yu, Rui Zhang, Stanley C. K. Lau

**Affiliations:** a Division of Life Science, the Hong Kong University of Science and Technology, Clear Water Bay, Hong Kong; b State Key Laboratory of Marine Environmental Science, Xiamen University, Xiamen, Fujian, China

## Abstract

Recent findings of *Escherichia coli* persisting autochthonously in environmental matrices outside animal bodies have revealed largely unknown facets of the lifestyle and ecophysiology of the species that have yet to be explored. Here, we report the draft genome sequence of *E. coli* E1728 isolated from marine sediment.

## GENOME ANNOUNCEMENT

*Escherichia coli* naturally inhabits the lower intestines of warm-blooded animals. Once egested into the external environment, it is faced with a large number of stresses that are different from those inside the animal hosts. Therefore, it was commonly believed that *E. coli* cannot survive for long in the external environment. However, many recent studies have reported *E. coli* populations that persisted autochthonously in soils and sediments without any known association with fecal contamination (1). It was estimated that on the global scale, half of the total population of naturally occurring *E. coli* exists outside animal hosts (2). Therefore, the study of *E. coli* as it thrives in external environments is essential to the understanding of the evolution and lifestyle of the species as a whole.

We isolated *E. coli* E1728 from the sediment of an intertidal mudflat in Hong Kong. Its genomic DNA was extracted using the Wizard genomic DNA purification kit (Promega, USA) and sequenced on an Illumina HiSeq 2000 system using paired-end libraries with 300-bp inserts. The raw reads were *de novo* assembled manually into contigs using the CLC Genomics Workbench version 6.5.1 (CLC bio, USA). A total of 170 contigs were obtained for E1728, ranging from 509 to 364,372 bp each (*N*_50_, 131,760 bp), with a total size of 5,264,979 bp and a G+C content of 50.3%. The number of protein-coding sequences (CDSs) predicted using GeneMarkS was 5,135 (3). The total length of the CDSs comprises 88.17% of the total contig size. Homologous comparison of the CDSs using BLAST identified 3,878 genes in 22 COG functional groups and 3,878 genes in 31 KEGG metabolic pathway groups. Using tRNAscan (4) and RNAmmer (5), E1728 was found to carry 79 tRNAs and 3 rRNAs.

### Nucleotide sequence accession numbers.

This whole-genome shotgun project has been deposited in DDBJ/EMBL/GenBank under the accession no. JDFV00000000. The version described in this paper is the first version, JDFV01000000.
